# The modulation of Dicer regulates tumor immunogenicity in melanoma

**DOI:** 10.18632/oncotarget.10273

**Published:** 2016-06-23

**Authors:** Nicholas C. Hoffend, William J. Magner, Thomas B. Tomasi

**Affiliations:** ^1^ Laboratory of Molecular Medicine, Department of Immunology, Roswell Park Cancer Institute, Buffalo, New York, USA; ^2^ Department of Medicine, State University of New York, Buffalo, New York, USA; ^3^ Department of Microbiology and Immunology, School of Medicine and Biomedical Sciences, State University of New York, Buffalo, New York, USA

**Keywords:** Dicer, microRNA, melanoma, immunogenicity, CD8^+^ T cells

## Abstract

MicroRNAs (miRs) are small non-coding RNAs that regulate most cellular protein networks by targeting mRNAs for translational inhibition or degradation. Dicer, a type III endoribonuclease, is a critical component in microRNA biogenesis and is required for mature microRNA production. Abnormal Dicer expression occurs in numerous cancer types and correlates with poor patient prognosis. For example, increased Dicer expression in melanoma is associated with more aggressive tumors (higher tumor mitotic index and depth of invasion) and poor patient prognosis. However, the role that Dicer plays in melanoma development and immune evasion remains unclear. Here, we report on a newly discovered relationship between Dicer expression and tumor immunogenicity. To investigate Dicer's role in regulating melanoma immunogenicity, Dicer knockdown studies were performed. We found that B16F0-Dicer deficient cells exhibited decreased tumor growth compared to control cells and were capable of inducing anti-tumor immunity. The decrease in tumor growth was abrogated in immunodeficient NSG mice and was shown to be dependent upon CD8^+^ T cells. Dicer knockdown also induced a more responsive immune gene profile in melanoma cells. Further studies demonstrated that CD8^+^ T cells preferentially killed Dicer knockdown tumor cells compared to control cells. Taken together, we present evidence which links Dicer expression to tumor immunogenicity in melanoma.

## INTRODUCTION

Immunotherapeutic strategies for treating cancer have become more prevalent over the past few years [1]. Several hurdles must be overcome for successful immunotherapy treatments. One such hurdle involves the tumor's immunogenicity and the immune cell's ability to properly recognize and destroy them [2]. Tumors undergo immune escape by losing antigen expression, acquiring defects in antigen processing/presentation, and/or inducing immunosuppressive molecules [3]. Melanoma cells can undergo immune escape through various mechanisms involving immune gene regulation [4–8]. Immunotherapy has achieved some success in certain subsets of melanoma patients; however, the regulation of immune effector molecules important for melanoma immunogenicity remains poorly understood [9, 10]. Recent work has demonstrated that microRNAs (miRs) may be involved in modulating tumor immunogenicity.

MicroRNAs are ~22 nucleotide non-coding RNAs that fine-tune gene expression post-transcriptionally [11]. The biogenesis of miRs is tightly regulated by diverse and evolutionarily conserved factors including Dicer, an RNAse III enzyme [12]. Dicer is responsible for cleaving the pre-miR into a mature miR duplex in the cytoplasm and is required for most miR biogenesis [13]. Upon completion of miR maturation, the mature miR duplex is unwound and the guide strand is loaded onto the RNA induced silencing complex (RISC) [12]. Dicer is essential for proper embryonic development/organogenesis in vertebrates. An embryonic knockout of Dicer is lethal in mice [14]. Recent work has shown that Dicer plays a substantial role in the development and function of immune cells [15]. Mutations in Dicer are present in several types of cancer as well as other diseases [16]. For example, DICER1 mutations have been described as a factor in pleuropulmonary blastoma and pituitary blastoma [17, 18]. Additionally, a potential role for Dicer in oncogenesis was identified by the observation that diminished miR processing augmented tumorigenesis and cellular transformation in lung cancer [19]. Moreover, several cancer subtypes possess abnormal Dicer expression, correlating with poor patient prognosis [20]. For example, Dicer expression is high in specific subsets of melanoma and correlates with more advanced tumors/poor patient prognosis, while other subsets of melanoma have low Dicer expression that correlates with more advanced tumors/poor patient prognosis [21–23].

Bioinformatics studies suggest that miRs may preferentially target immune genes [24]. Immuno-miRs were defined as a subset of miRs that have the ability to regulate several immune cell processes [24]. For instance, immuno-miRs have been implicated in T cell development, differentiation, activation, and function [25]. Immuno-miRs also play important roles in B cell biology [26]. Independent of the immune system, Dicer-dependent miRs can alter tumor cell immunogenicity. For example, in a glioma model, miR-222 and miR-339 were demonstrated to target and inhibit ICAM-1 expression, thus diminishing tumor sensitivity to lysis by cytotoxic T cells [27]. Also, miR-34a and miR-34c both target ULBP2, a natural killer (NK) cell ligand for the NKG2D receptor [28]. Expression of these two miRs in human melanoma cell lines was reported to decrease ULBP2 surface expression and result in less recognition/killing by NK cells [28]. A landmark study reported numerous miRs that target and modulate expression of human NKG2D ligands [29]. MiR-17-5p, miR-20a, miR-93, miR-106b, miR-373, and miR-520 all target MICA or MICB, two ligands of the NKG2D receptor, and regulate their expression in several human cancer cell lines [29]. This modulation by miRs affected NK cell recognition as well as lysis of tumor cells *in vivo* [29]. MiR-10b also targets MICB and promotes resistance of human cancer cell lines to NK cell mediated lysis [30]. Collectively, these studies demonstrate that changes in miR expression can alter tumor immunogenicity and promote cell-mediated immune responses. However, the effect of Dicer expression on melanoma tumor immunogenicity remains largely unknown.

The studies reported here were designed to determine whether alterations in melanoma expression of Dicer protein were capable of alleviating an aggressive tumor phenotype and enhancing tumor control. We examined the consequence of Dicer knockdown on melanoma cell proliferation, tumor growth kinetics, and overall survival of mice in two melanoma models. Also, we identified the contribution of specific immune cells in controlling the tumor growth of Dicer knockdown melanoma cells.

## RESULTS

**Figure 1:**
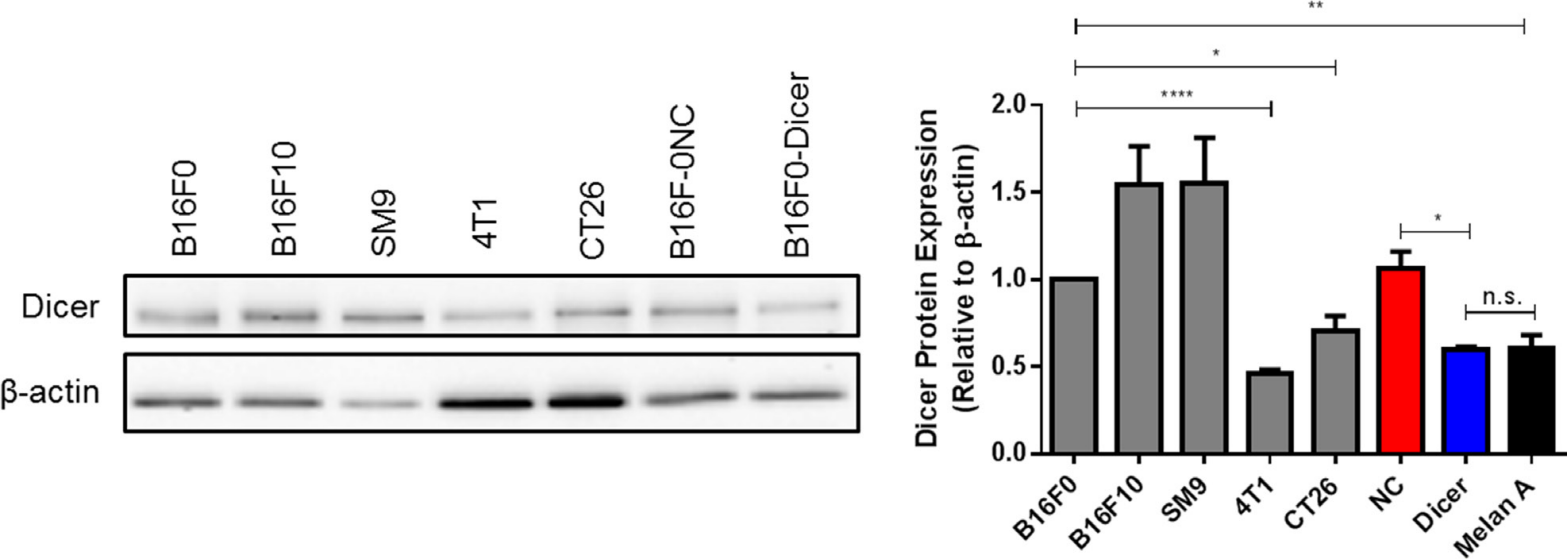
Murine melanoma cell lines express substantial levels of Dicer protein Various mouse cell lines harvested at equal confluency were used for preparation of total protein lysates and western blots were performed probing for Dicer and β-actin. B16F0 pGIPZ transduced cells were compared for Dicer expression to the cell lines and Melan A cells as well. Left panel - representative western blot, right panel - quantification of multiple biological and technical replicates. Error bars ± SEM, *p < .05, **p < .005, ****p < .0005.

Cutaneous melanomas with poor prognosis are associated with high Dicer expression [22, 23]. The Dicer expression of the B16 murine model of cutaneous melanoma was compared to other murine models, including SM9 trophoblast, 4T1 mammary carcinoma, and CT26 colon carcinoma. Consistent with published reports in human melanoma, Dicer expression in B16F0 (non-metastatic) and B16F10 (metastatic) was significantly higher compared to the 4T1 and CT26 cell lines and was also significantly elevated relative to normal murine melanocytes (Melan A) (Figure 1) [22, 23]. Upon verification that B16F0 and B16F10 have high Dicer expression, we used these cell lines to further study the effects of Dicer on primary tumor growth in cutaneous melanoma. To determine if altering Dicer expression affected melanoma tumor growth, B16F0 cells were transduced with the pGIPZ lentiviral vector encoding either a non-silencing shRNA (B16F0-NC) or an shRNA targeting Dicer (B16F0-Dicer). Initial studies investigated the knockdown efficiency of the shRNA-Dicer clone used. We found a ~50% reduction in Dicer protein expression compared to the control non-silencing shRNA (Figure 1). Additionally, the B16F0-Dicer cells had similar Dicer protein expression compared to normal mouse melanocytes (Melan A) and the low Dicer expressing breast cancer cell line 4T1 (Figure 1). These results suggest that Dicer is significantly enhanced in melanoma compared to normal mouse melanocytes and that our Dicer knockdown model does not ablate/delete Dicer expression, but rather restores it to a more homeostatic level.

**Figure 2:**
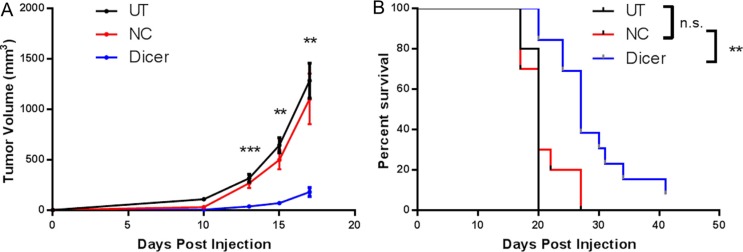
Diminished Dicer protein expression in B16F0 decreases tumor growth *in vivo* (**A**) C57BL/6 mice were challenged subcutaneously with 10^5^ untreated B16F0 cells (UT, *n* =10), B16F0 non-silencing shRNA cells (NC, *n* = 10) or B16F0 Dicer shRNA cells (Dicer, *n* = 13). Data are plotted as average tumor volume. (**B**) Percent survival of mice from (**A**) as plotted against days post injection of tumor cells. Error bars ± SEM, *p < .05, **p < .005, ***p < .0005.

Upon confirmation of stable Dicer knockdown, C57BL/6 mice were challenged subcutaneously with B16F0, B16F0-NC, or B16F0-Dicer and tumor growth/survival was monitored (Figure 2A). There was no significant difference between the growth of B16F0 tumors and B16F0-NC tumors. However, the B16F0-Dicer tumors grew significantly slower than the B16F0-NC tumors. Mice challenged with B16F0-Dicer had significantly longer overall survival than mice challenged with B16F0-NC (Figure 2B). This data suggests that manipulating Dicer expression in melanoma can alter tumor growth.

**Figure 3:**
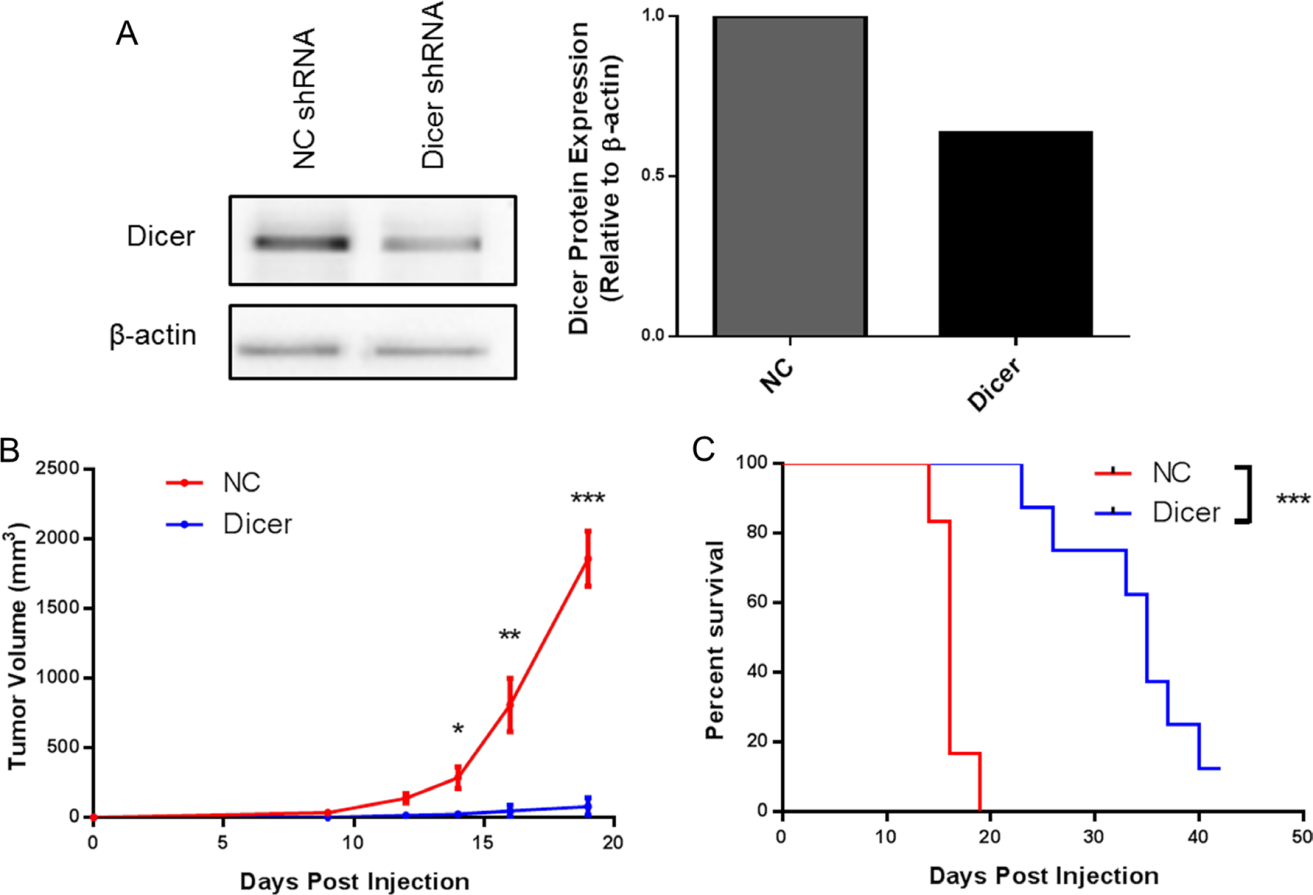
Diminished Dicer protein expression in B16F10 decreases tumor growth *in vivo* (**A**) B16F10 pGIPZ shRNA transduced cells were harvested and probed for Dicer protein expression via western blot. (**B**) C57BL/6 mice were challenged subcutaneously with 10^5^ B16F10 non-silencing shRNA cells (NC, *n* = 6) or B16F10 Dicer shRNA cells (Dicer, *n* = 8). Data are plotted as average tumor volume. (**C**) Percent survival of mice from (**B**) as plotted against days post injection of tumor cells. Error bars ± SEM, *p < .05, **p < .005, ***p < .0005.

Our finding that Dicer knockdown delays tumor growth in melanoma was extended to a second model, the metastatic B16F10 melanoma line. B16F10 cells were transduced with the pGIPZ lentiviral vector encoding either a non-silencing shRNA (B16F0-NC) or shRNA targeting Dicer (B16F0-Dicer), followed by assessment of Dicer protein knockdown. Similar to the B16F0 transduced cells, there was a ~40% reduction in Dicer protein expression compared to the non-silencing shRNA (Figure 3A). C57BL/6 mice were challenged subcutaneously with B16F10-NC or B16F10-Dicer and tumor growth/survival monitored (Figure 3B). In agreement with our earlier findings, the B16F10-Dicer tumors grew significantly slower compared to the B16F10-NC tumors. Furthermore, the mice challenged with B16F10-Dicer had significantly longer overall survival compared to B16F0-NC (Figure 3C). Thus, in both metastatic and non-metastatic mouse cutaneous melanoma cell lines, reduced Dicer expression was associated with decreased tumor growth and enhanced survival. Because B16F0-Dicer and B16F10-Dicer behaved similarly, subsequent studies focused on primary tumor growth in the absence of metastasis using B16F0-Dicer.

**Figure 4:**
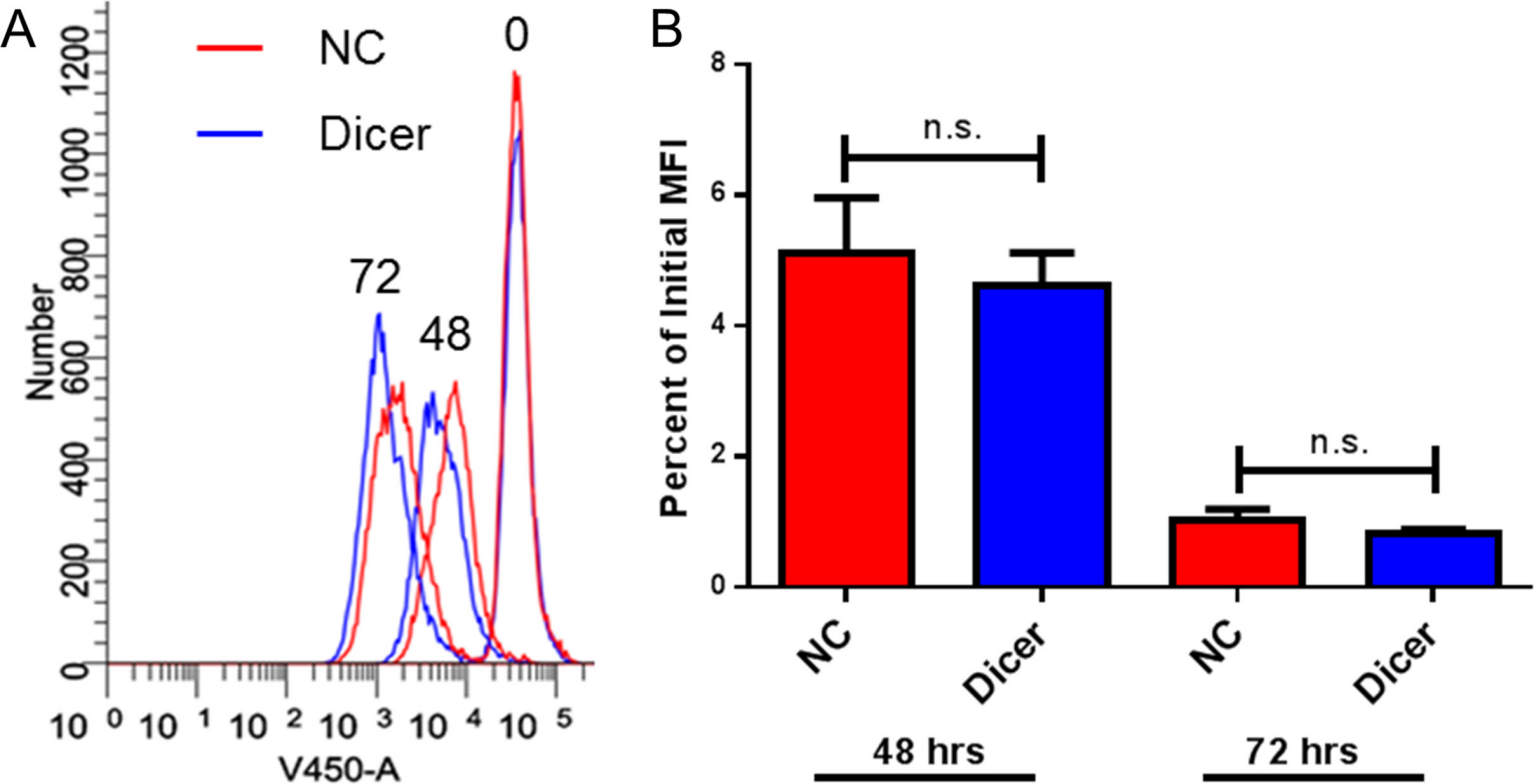
Down-regulation of Dicer does not affect proliferation in B16 melanoma cells B16F0-NC and B16F0-Dicer cells were labeled with either Cell Trace Far Red or Cell Trace Violet and cultured for 48–72 hours. The cells were harvested and analyzed by flow cytometry. (**A**) Representative histogram of the three time points. (**B**) Three independent experiments were compiled and quantified by the percent dye dilution of the 48–72 hour samples compared to the time zero dye staining. Error bars ± SEM.

Previous work demonstrated that altering Dicer expression in cancer cell lines can affect their proliferation rates *in vitro* [31, 32]. We therefore hypothesized that Dicer knockdown in melanoma cells could affect their proliferation and result in delayed tumor growth *in vivo*. To assess cell proliferation in our system, a fluorescent dye dilution assay was performed. B16F0-NC and B16F0-Dicer cells were fluorescently labeled, cultured for 48–72 hours, and flow cytometric analysis was performed. There was no difference in proliferation between the B16F0-NC and B16F0-Dicer cells, as measured by dye dilution (Figure 4A and 4B). Therefore, we conclude that the delayed tumor growth of B16F0-Dicer *in vivo* was not due to decreased cell proliferation.

**Figure 5:**
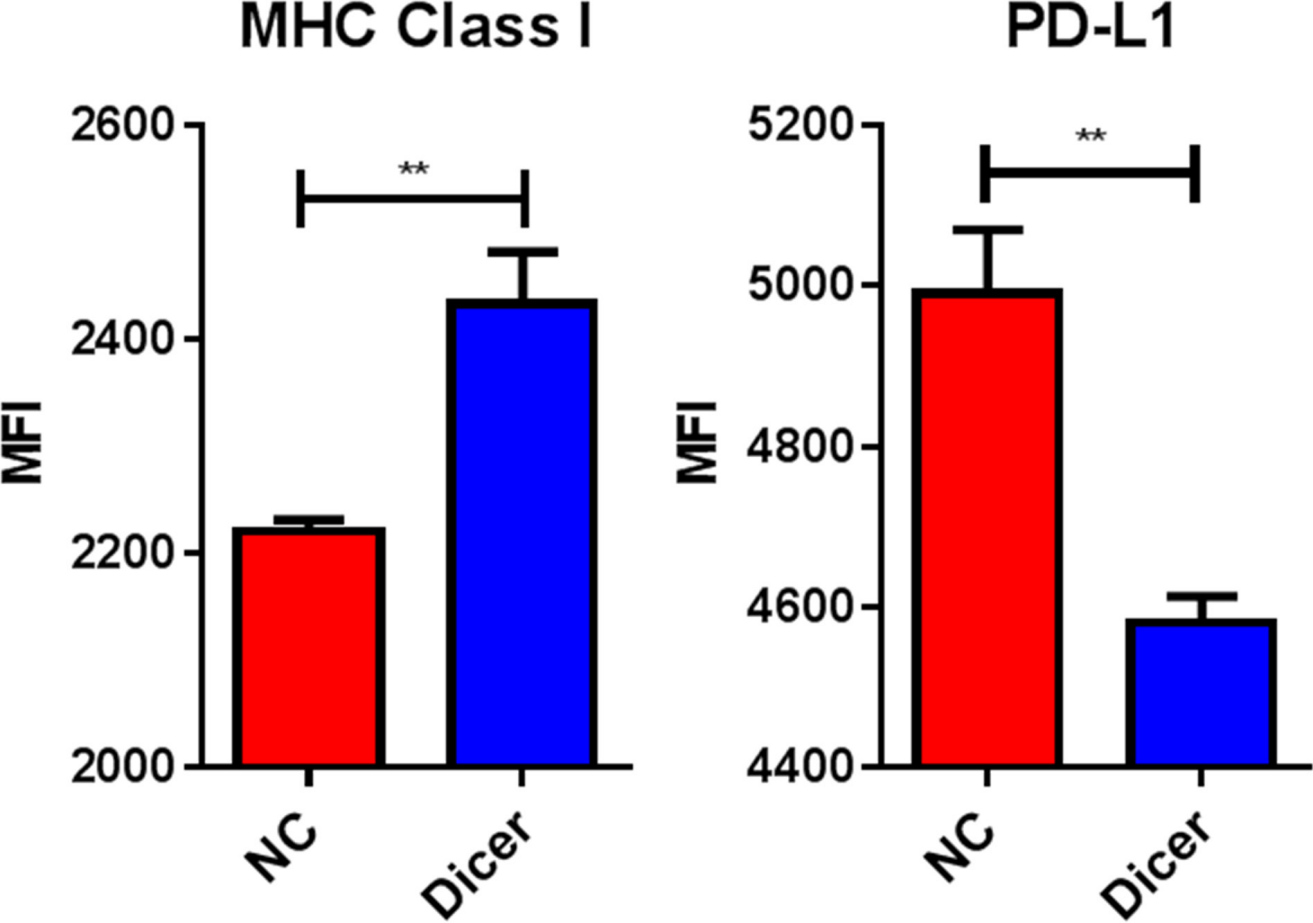
Dicer knockdown induces a more immunogenic cellular phenotype B16F0-NC and B16F0-Dicer cells were treated with 250 U/mL mouse IFNγ for 24 hours and probed for cell surface expression of MHC Class I (H-2D^b^) and PD-L1 via flow cytometry. Error bars ± SEM, **p < .005.

Since reduced Dicer expression led to decreased tumor growth without altered tumor cell proliferation and miRs are known to target various immune genes, we hypothesized that B16F0-Dicer and B16F10-Dicer may have enhanced immunogenicity. We therefore assessed cell surface expression of several immunomodulatory proteins by treating B16F0-NC and B16F0-Dicer cells with 250 U/mL IFNγ for 24 hours. The B16F0-NC cells had significantly less MHC Class I induced compared to the B16F0-Dicer cells (Figure 5). Furthermore, the B16F0-NC cells had significantly higher levels of the immunoinhibitory molecule PD-L1 induced compared to the B16F0-Dicer cells (Figure 5). This demonstrates that Dicer knockdown promotes a more immunogenic and responsive immune gene profile on melanoma cells.

**Figure 6:**
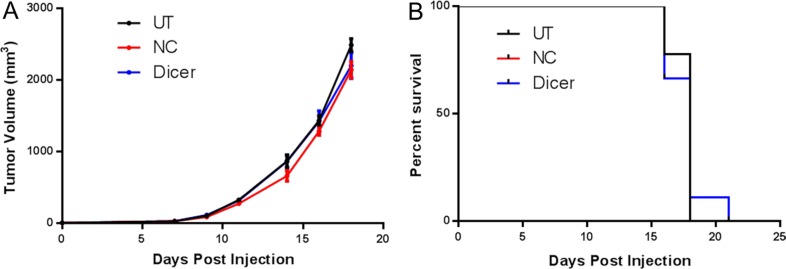
A functional immune system is required for the decreased growth of B16F0 Dicer knockdown tumors (**A**) NSG mice were challenged with 10^5^ untreated B16F0 cells (UT, *n* = 9), B16F0 non-silencing shRNA cells (NC, *n* = 9), or B16F0 Dicer shRNA cells (Dicer *n* = 9). Data are plotted as average tumor volume. (**B**) Percent survival of mice from (**A**) as plotted against days post injection of tumor cells. Error bars ± SEM.

Because the B16F0-NC and B16F0-Dicer cells showed comparable proliferation and the B16F0-Dicer cells had a more immunogenic phenotype, we hypothesized that an anti-tumor immune response was responsible for the delayed tumor growth *in vivo*. NSG mice lack functional CD4^+^, CD8^+^, and NK cells and also contain defects in components of the innate immune system and cytokine signaling [33]. We challenged NSG mice with B16F0, B16F0-NC, or B16F0-Dicer to determine whether a functional immune response was required for the delayed tumor growth of B16F0-Dicer. Strikingly, the delay in tumor growth by Dicer knockdown was abrogated in the immunodeficient mice. There was no significant difference in either the tumor growth or survival of mice challenged with B16F0-NC or B16F0-Dicer (Figure 6A and 6B). Our data suggest that the impact of Dicer knockdown on melanoma tumor growth was dependent upon an anti-tumor immune response. Additionally, these observations further demonstrate that Dicer knockdown did not induce a proliferative defect since all tumors grew with similar kinetics *in vivo*.

**Figure 7:**
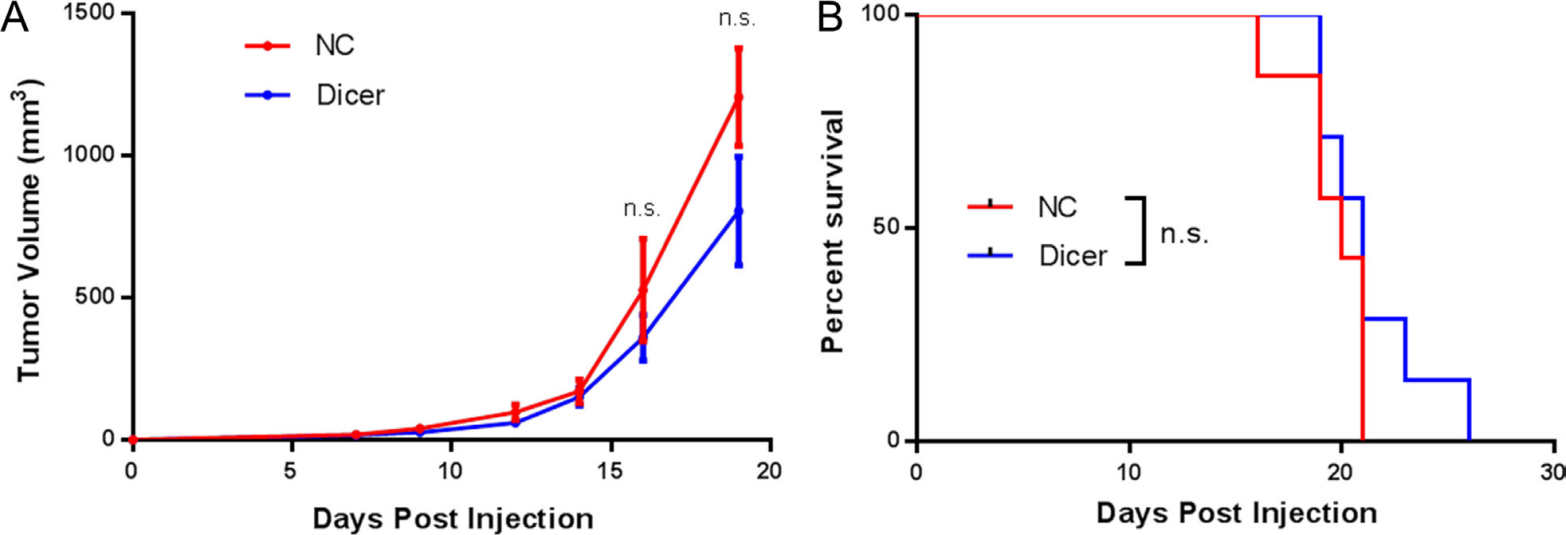
CD8^+^ T cells are required for the decreased growth of B16F0 Dicer knockdown tumor cells (**A**) CD8^−/−^ mice were challenged with 10^5^ B16F0 non-silencing shRNA cells (NC, *n* = 7) or B16F0 Dicer shRNA cells (Dicer, *n* = 7). Data are plotted as average tumor volume. (**B**) Percent survival of mice from (**A**) as plotted against days post injection of tumor cells. Error bars ± SEM.

The differential tumor growth of B16F0-Dicer cells in C57BL/6 mice compared to NSG mice suggested an anti-tumor immune response was occurring. We therefore investigated the role of CD8^+^ T cells in this response. CD8^+^ T cells are potent killers of tumor cells in melanoma and therefore would be expected to be involved in controlling the growth of B16F0-Dicer tumors [34]. CD8^−/−^ mice were challenged subcutaneously with B16F0-NC or B16F0-Dicer and tumor growth/survival was monitored. There was no significant difference in either the tumor growth or survival of mice challenged with B16F0-NC or B16F0-Dicer. This demonstrates that CD8^+^ T cells are one of the major immune cell populations mediating the anti-tumor immune response to the B16F0-Dicer cells. (Figure 7A and 7B).

**Figure 8:**
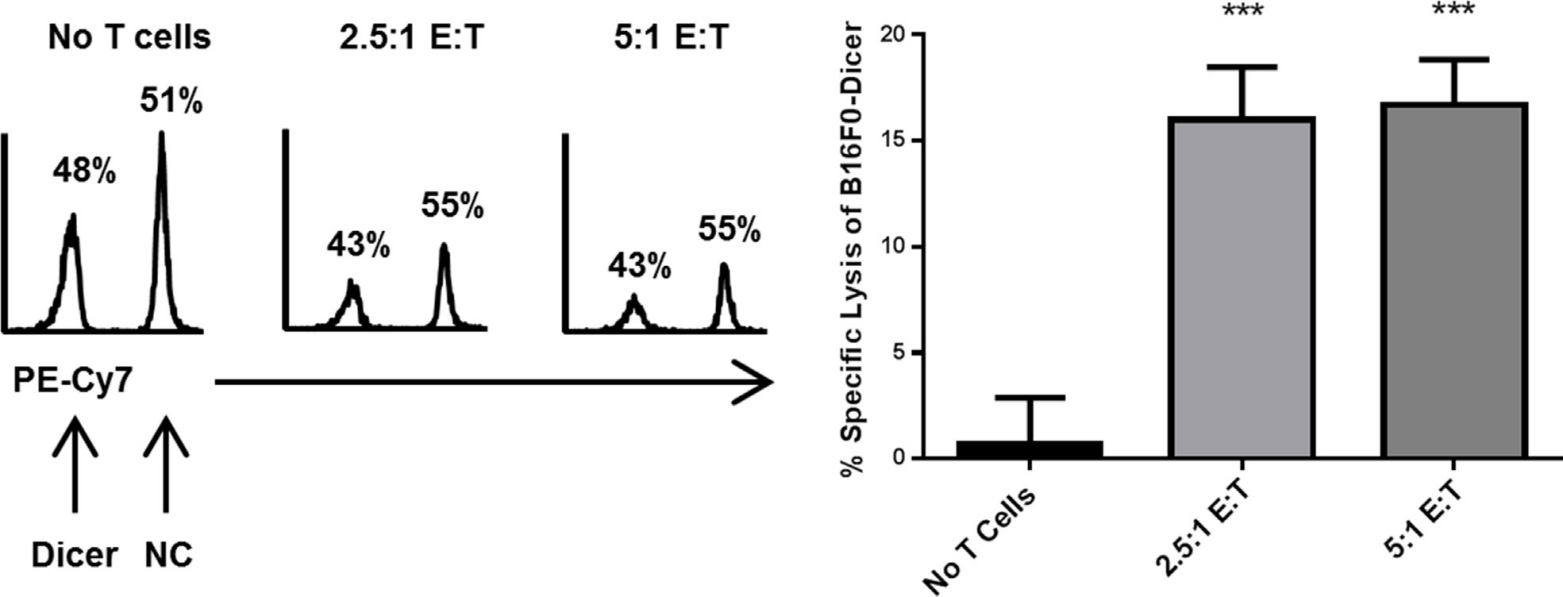
Dicer knockdown in melanoma enhances CD8^+^ T cell cytotoxicity Effector Pmel-1 CD8^+^ cells were cultured for 24 hours at varying ratios with a 1:1 mixture of B16F0-NC labeled with Cell Trace Far Red and B16F0-Dicer labeled with Cell Trace Violet. Following culture, the cells were analyzed by flow cytometry. Left, representative histograms of target cells. Right, percent specific lysis of B16F0-Dicer target cells. Data are from three independent experiments. Error bars ± SEM. ***p < .0005.

Little is known regarding how changes in Dicer expression can alter tumor cell susceptibility to CD8^+^ T cell cytotoxicity. Since CD8^+^ T cells are required for the anti-tumor response to B16F0-Dicer, we hypothesized that it was due to enhanced lysis by CD8^+^ T cells. To test this, splenocytes were isolated from pmel-1 transgenic mice, which have a T cell receptor specific for the melanoma gp100 antigen [35, 36]. The splenocytes were primed/activated to generate effector CD8^+^ T cells. Following activation, the splenocytes were co-cultured with a 1:1 mix of B16F0-NC (labeled with Cell Trace Far Red) and B16F0-Dicer (labeled with Cell Trace Violet) for 24 hours at increasing effector to target ratios. We observed ~ 1:1 ratio of B16F0-NC to B16F0-Dicer cells in the no T cells cultures (Figure 8). Importantly, there was a significant decrease in the ratio of B16F0-Dicer cells to B16F0-NC cells when activated T cells were added demonstrating a preferential killing of the B16F0-Dicer cells by CD8^+^ T cells (Figure 8). Gp100 antigen expression was similar between the B16F0-Dicer and B16F0-NC cells, implicating altered susceptibility to CD8^+^ T cell lysis due to immune gene regulation (data not shown). The ratio of T cells used did not change the B16F0-Dicer specific lysis, suggesting that Dicer knockdown melanoma cells have increased susceptibility to CD8^+^ T cell lysis through their enhanced antigen presentation and weakened ability to induce PD-L1 mediated T cell exhaustion.

## DISCUSSION

MicroRNA dysregulation is common in multiple diseases and research into the miR biogenesis machinery has been an area of intense investigation [16, 18, 20, 37–49]. Previous studies from our lab demonstrated that treatment of melanoma with HDACi elicited durable and specific anti-tumor immunity, while concurrently decreasing tumor expression of Dicer and increasing immune gene expression [50, 51]. The current study was initiated to determine if the anti-tumor effects of HDACi could be recapitulated by manipulating Dicer expression. Dicer knockdown in B16F0 cells was able to partially recapitulate the effects of HDACi treatment, suggesting that Dicer and miRs are involved, but additional pathways are required in the HDACi induced anti-tumor immune response. A functional immune system is vital for the anti-tumor effects of HDACi treatment [52]. This potentially links the HDACi regulation of Dicer expression/anti-tumor immunity to the observation that delayed tumor growth of B16F0-Dicer was abrogated in NSG and CD8^−/−^ mice. To our knowledge, Dicer expression has not been reported as a regulator of immunogenicity. We hypothesize that tumor cells may undergo immune escape by dysregulating Dicer expression, suggesting a potential role and explanation for the abnormal Dicer expression in various cancers. For example, a recent report demonstrated that B cells from patients with multiple sclerosis exhibited decreased Dicer expression that resulted in enhanced expression of CD80, suggesting Dicer may be pertinent in immune cell function in certain disease states [53]. Additionally, our lab has found reduced Dicer expression in multiple sclerosis patient PBLs and showed that Dicer levels increased after IFNβ_1a_ therapy [49]. Here, we provide a new link between Dicer expression and tumor immunogenicity.

Recent literature evidence suggests that melanoma and colorectal cancer possess high Dicer expression that correlated with poor patient prognosis, while low Dicer expression in breast cancer correlated with poor patient prognosis [22, 23, 54]. Several groups have investigated Dicer in a wide range of human patient tumor samples and correlated expression levels with several factors including disease progression and overall patient survival [20]. Interestingly, some of these reports contain conflicting results on the identical cancer types. Factors affecting the data could include prior treatments the patients had received, differences in diet and exercise, handling and processing of tumor samples, different laboratory reagents used in conducting the experiments, and the method of determining Dicer expression. Ma *et al.* [23] demonstrated that cutaneous and acral lentiginous melanoma subtypes had high Dicer expression that correlated with more advanced tumors and poor patient prognosis, while mucosal and desmoplastic melanoma subtypes had low Dicer expression that correlated with more advanced tumors and poor patient prognosis. In contrast, Jafarnejad *et al.* [21] showed that melanoma had low Dicer expression that correlated with poor patient prognosis. However, in their study all melanoma subtypes were grouped into either primary or metastatic melanomas, potentially obscuring the differences Ma *et al.* [23] reported. Currently, a gap in knowledge of Dicer biology exists because most studies have only correlated Dicer expression in patient tumor samples to various outcomes without mechanistic connections. Also, few studies have dissected Dicer's role in tumor development, progression, or immune escape. These gaps leave much to be investigated in regard to Dicer's role in cancer and immune responses. Our studies utilizing the B16 cutaneous melanoma model did however allow the dissection of Dicer's role in melanoma progression and immune evasion.

In this study, we report that B16F0-Dicer cells have a more responsive immune gene profile when treated with IFNγ compared to control cells (higher MHC Class I and lower PD-L1). Future work should evaluate whether other immunomodulatory cytokines similarly affect B16F0-Dicer cells. Evaluating patient samples to correlate their immune gene expression with Dicer levels could strengthen these studies. Additionally, it will be important to determine what miRs are changing due to the Dicer knockdown and the resulting effect on tumor cell immune gene expression. Determining if Dicer knockdown sensitizes melanoma cells to chemotherapy, radiation, adoptive cell transfer, immune checkpoint inhibitors, or imiquimod may strengthen the notion of targeting Dicer expression in melanoma prior to or in combination with current treatment strategies. These studies may provide a more direct link between the regulation of Dicer and changes in tumor expression of immune genes/immunogenicity, while also promoting Dicer expression as a target in clinical settings.

Future studies to determine if Dicer affects immunogenicity in additional tumor types where high Dicer expression is unfavorable, including colorectal and prostate, could show a role for Dicer in regulating the immunogenicity of other cancers. However, in cancers where Dicer expression is low, such as in breast and ovarian cancer, it would be important to determine if restoring Dicer expression to normal levels could recapitulate the effects of the studies shown here. Dicer is not the only factor involved in microRNA biogenesis that can be dysregulated in disease states. Complementary members of the miR biogenesis pathway, including Drosha, DGCR8, and Argonaute-2, have been implicated in cancer [38, 42, 55–58]. Expanding future experiments to include additional members of the miR biogenesis pathway may also provide information regarding how and why tumors dysregulate miRs and successfully escape immune detection.

Collectively, we have demonstrated that (1) Dicer knockdown in melanoma decreased tumor progression and extended overall survival of mice; (2) melanoma cells with Dicer knockdown induced an anti-tumor immune response; (3) control of Dicer knockdown melanoma tumors was dependent upon an intact immune system, especially CD8^+^ T cells; and (4) melanoma cell over-expression of Dicer weakened CD8^+^ T cell recognition/killing.

## MATERIALS AND METHODS

### Animals and cell culture

All experiments using animals were performed according to protocols approved by RPCI/IACUC. Upon delivery, mice were housed in the Division of Laboratory Animal Resources (DLAR) at RPCI in a pathogen-free barrier facility and water/food was freely available at all times. 6–8 week old male and female C57BL/6NCr mice were purchased from NCI and Charles River. 6–12 week old NSG (NOD.Cg-Prkdc^*scid*^ *Il2rg*^*tm1Wjl*^/SzJ) male and female mice were provided by the RPCI DLAR colony. 5–6 week old female CD8^−/−^ (B6.129S2-*Cd8a^tm1Mak^*/J) mice were purchased from Jackson Laboratories. 8–12 week old male pmel-1 TCR transgenic mice (B6.Cg-*Thy1*^a^/Cy Tg (TcraTcrb)8Rest/J) were purchased from Jackson Laboratories. The mouse melanoma B16F0 and B16F10, mouse colon carcinoma CT26, and mouse mammary carcinoma 4T1 cell lines were purchased from American Type Culture Collection (ATCC) and cultured according to ATCC's instructions. The mouse trophoblast SM9 was a gift from Joan Hunt. Mouse IFNγ was purchased from R&D Systems for use in *in vitro* cultures.

### Generation of stable Dicer knockdown cell lines

B16F0 or B16F10 cells were transduced using the pGIPZ lentiviral vector encoding non-silencing shRNA or Dicer shRNA (Thermo Fisher Open Biosystems). Stably transduced cells were established by puromycin selection. Throughout all experiments, the transduced cells were kept under puromycin selection to ensure stable shRNA expression. The efficiency of shRNA knockdown was routinely measured at the protein level by western blots. These cell lines are termed B16F0-NC (non-silencing shRNA), B16F0-Dicer (Dicer shRNA), B16F10-NC (non-silencing shRNA), and B16F10-Dicer (Dicer shRNA).

### Western blotting

For whole protein extracts, cells were harvested, pelleted at 450 × g for 7 minutes, and washed once with Phosphate Buffered Saline (PBS) (Life Technologies). The pellets were lysed on ice for 60 minutes with RIPA lysis buffer (Sigma-Aldrich) supplemented with protease inhibitor cocktail (Sigma-Aldrich), phosphatase inhibitor cocktail (Thermo Scientific), and 1 mM dithiothreitol (Sigma-Aldrich). The extract was then centrifuged at 10,000 × g for 10 minutes and supernatants collected. Protein concentrations were determined with the Micro BCA Assay Kit (Pierce). 20 μg of protein lysates were heated for 5 minutes at 95°C in SDS sample buffer plus 0.13 M dithiothreitol (Sigma-Aldrich), separated on 7% [for Dicer] or 15% [for β-actin] SDS-PAGE gels and transferred to Immun-blot LF PVDF membranes (BioRad). Membranes were blocked using 5% non-fat dry milk followed by addition of primary antibodies. Antibodies used were anti-Dicer (Cat # A301-936A, Bethyl Laboratories), anti-β-actin (Cat # 8227, Abcam), and goat anti-rabbit IgG-horseradish peroxidase (Cat # W4011, Promega). Blots were developed with a West Pico Chemiluminescent Kit (Pierce) and imaged with a Chemidoc MP imager (BioRad).

### Flow cytometry

Flow cytometric analysis was conducted by standard methods with cells fixed with 1% paraformaldehyde and run on a BD LSR2 flow cytometer (BD Biosciences); compensation and analysis were performed using Winlist 7.1. R-Phycoerythrin (PE) conjugated anti-H-2D^b^ and isotype control were from BD Pharmingen. R-Phycoerythrin (PE) conjugated anti-PD-L1 was from eBiosciences.

### *In vitro* proliferation assay

B16F0-NC and B16F0-Dicer cells were fluorescently labeled with Cell Trace Far Red (Life Technologies) or Cell Trace Violet (Life Technologies) according to the manufacturer's instructions. The cells were then cultured for 48–72 hours and analyzed by flow cytometry.

### Pmel-1 CD8^+^ T cells

Spleens were harvested from 16–20 week old male pmel-1 mice, mechanically disrupted, filtered through a 70 μm filter (BD Biosciences), washed with PBS, and centrifuged at 450 × g for 5 minutes. The pellet was re-suspended in ACK lysis buffer for 2 minutes to lyse the red blood cells, filtered through a 70 μm filter, washed with PBS, and centrifuged at 450 × g for 5 minutes. The splenocytes were then cultured in one of two ways, 1) with 0.1 ug/mL gp100_25–33_ peptide and 30 IU/mL rIL-2 (Sigma-Aldrich) for 5 days, 2) with CD3/CD28 beads (Thermo Fisher) and 30 IU/mL rIL-2 for 5 days, in RPMI 1640 containing 10% FBS, 2-mercaptoethanol, nonessential amino acids, sodium pyruvate, and penicillin/streptomycin at 37°C in 5% CO_2_. Flow cytometric analysis confirmed a CD8^+^ T cell effector phenotype prior to use in the cytotoxicity assay.

### *In vitro* cytotoxicity assay

B16F0-NC cells were fluorescently labeled with Cell Trace Far Red and B16F0-Dicer cells were fluorescently labeled with Cell Trace Violet according to the manufacturer's instructions. The two cell types were mixed together at a 1:1 ratio and cultured with or without activated Pmel-1 CD8^+^ T cells for 24 hours. The labeled tumor cells were analyzed by flow cytometry. The proportion of fluorescently-labeled cells was plotted as PE-Cy7^+^ (B16F0-NC) and PE-Cy7^−^ (B16F0-Dicer) cells on histograms, and the percent specific lysis of the B16F0-Dicer cells was determined by the formula [1-(% PE-Cy7^−^ cells / % PE-Cy7^+^ cells)] × 100%.

### Tumor injections and assessment of growth

10^5^ untreated B16F0, B16F0 transduced with pGIPZ lentiviral shRNA, untreated B16F10, or B16F10 transduced with pGIPZ lentiviral shRNA were injected subcutaneously into the right flank of mice. Tumor growth was measured three times weekly and the volumes were calculated using the formula *(width*^2^ *× length)/*2. Mice were euthanized once tumors reached 1500mm^3^.

### Statistical analysis

Statistical analyses were performed with GraphPad version 6.05 software. Results are shown as mean ± SEM. Comparison of results from western blots, proliferation assays, cytotoxicity assays, or tumor volumes were carried out using the two-tailed unpaired *t*-test. Kaplan-Meier survival curves were compared using the Mantel-Cox log rank test. *p < .05, **p < .005, ***p < .0005.

## Abbreviations

miRs: microRNAs
RISC: RNA induced silencing complex
NK: natural killer
IFN: interferon
PBL: peripheral blood leukocytes
